# Alterations in Renin–Angiotensin System (RAS) Peptide Levels in Patients with HIV

**DOI:** 10.3390/metabo13010061

**Published:** 2022-12-31

**Authors:** Isaac Asante, Angela Lu, Brooks I. Mitchell, William A. Boisvert, Cecilia M. Shikuma, Dominic C. Chow, Stan G. Louie

**Affiliations:** 1 Department of Ophthalmology, Keck School of Medicine, University of Southern California, Los Angeles, CA 90033, USA; 2 Alfred Mann School of Pharmacy and Pharmaceutical Sciences, University of Southern California, Los Angeles, CA 90089, USA; 3 John A. Burns School of Medicine, University of Hawai’i at Manoa, Honolulu, HI 96813, USA

**Keywords:** renin-angiotensin system, metabolomics, carotid thickening, HIV

## Abstract

Chronic HIV infection has long been associated with an increased risk for cardiovascular diseases. The metabolites of the renin–angiotensin system (RAS) such as angiotensin II (AngII) play an important role in regulating blood pressure and fluid dynamics. Cross-sectional analysis of HIV-positive individuals (*n* = 71, age > 40 years, stable ART > 3 months with HIV viral load < 50 copies/mL) were compared to a similar HIV seronegative group (*n* = 72). High-resolution B-mode ultrasound images of the right carotid bifurcation (RBIF) and right common carotid artery (RCCA) were conducted to measure the extent of carotid atherosclerotic vascular disease. Plasma RAS peptide levels were quantified using a liquid chromatography-mass spectrometry-based metabolomics assay. RAS peptide concentrations were compared between persons with HIV and persons without HIV, correlating their association with clinical and cardiac measures. Median precursor peptides (Ang(1-12) and AngI) were significantly higher in the HIV-positive group compared to the HIV-negative. Analyses of the patient subgroup not on antihypertensive medication revealed circulating levels of AngII to be four-fold higher in the HIV-positive subgroup. AngII and TNF-alpha levels were found to have a positive association with RCCA, and AngI/Ang(1-12) ratio and TNF-alpha levels were found to have a positive association with RBIF. In both predictive models, AngIII had a negative association with either RCCA or RBIF, which may be attributed to its ability to bind onto AT2R and thus oppose pro-inflammatory events. These results reveal systemic alterations in RAS as a result of chronic HIV infection, which may lead to the activation of inflammatory pathways associated with carotid thickening. RAS peptide levels and cytokine markers were associated with RCCA and RBIF measurements.

## 1. Introduction

Cardiovascular disease (CVD) continues to be a major factor contributing to the morbidity associated with human immunodeficiency virus (HIV) even in the context of undetectable viral load [1]. HIV-infected individuals have a significantly increased risk for a myriad of CVD complications. These include increased risk for acute myocardial infarction, heart failure, peripheral arterial disease, and stroke [2]. Shah et al. have reported a 2.16-fold increase in risk for CVD in the HIV-positive population as compared to uninfected individuals [3]. This is further supported by other reports which have shown that HIV-positive status corresponded to a greater increased risk for CVD, similar to the risk that diabetes presents [4,5,6]. Furthermore, T cell activation and cytokine-driven inflammation are known risk factors for cardiovascular disease (CVD) [7].

**Figure 1:**
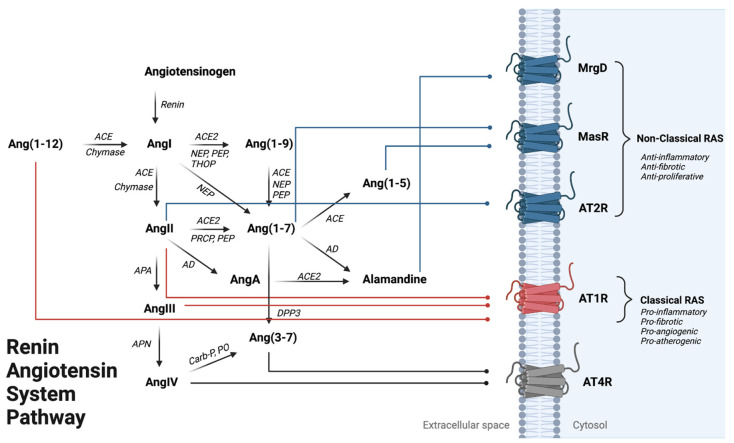
Renin–Angiotensin System Pathway Overview. Angiotensinogen is the penultimate RAS precursor that is cleaved by renin to form Angiotensin I (AngI). Alternatively, AngI can also be formed through chymase-mediated metabolism converting Ang(1-12) to AngI. AngI can be metabolized to Angiotensin II (AngII) through angiotensin-converting enzyme (ACE) or chymase-mediated metabolism, where AngII binding onto AT1R promotes the classical arm of RAS leading to vasoconstriction, inflammation activation, and cardiac hypertrophy. AngII can be further metabolized to form Angiotensin III (AngIII) by APA (aminopeptidase A), where AngIII metabolism via APN (aminopeptidase N) can form Angiotensin IV (AngIV), an AT4R agonist. Alternatively, AngII can form through NEP (neprilysin), PEP (prolyl endo-peptidase), and THOP (thimet oligopeptidase) mediated metabolism. Ang(1-7) is formed by (1) metabolism of AngII by ACE2, PEP, or PRCP (pro-X carboxypeptidase) or (2) an alternative pathway where Ang(1-9) can be metabolized by ACE, NEP, and PEP activity. Ang(1-7) can be metabolized to Ang(1-5) or Alamandine (AAng(1-7)) by ACE and AD (aspartate decarboxylase), respectively. AAng(1-7) can also be formed through AngII breakdown into AngA via AD, followed by AngA metabolism via ACE2. Ang(1-7) and Ang(1-5) can both bind onto MasR to counteract AT1R activation. AAng(1-7) binding onto MrgD, a Mas-related GPCR, can lead to vasodilation in the heart and kidney. Ang(3-7) can be formed by either (1) cleavage of AngIV by carboxypeptidase P (Carb-P) and prolyl oligopeptidase (PO) or by (2) dipeptidyl peptidase 3 (DPP3) cleavage of Ang(1-7). Red lines show the three RAS peptides that can being onto AT1R to activate the classical RAS pathway. Blue lines indicate peptide binding to anti-inflammatory signaling receptors AT2R, MasR, MrgD). Gray lines represent peptide binding onto the AT4R; the effects of this activation are currently not well delineated.

Although abnormal immune activation and ongoing inflammation have long been thought to contribute to increased CVD risk, the mechanisms linking HIV with CVD have yet to be fully elucidated. One potential pathway underlying carotid atherosclerosis may be through perturbation of the renin–angiotensin system (RAS), which is an important pathway regulating hemodynamics, fluid volume, and electrolyte homeostasis [8,9]. In addition, the RAS plays a pivotal role in regulating cell growth and differentiation, extracellular matrix metabolism (ECM), and chronic inflammation for various diseases [10,11,12,13]. Chronic activation of the “classical” or pathogenic arm of the RAS is mediated by angiotensin II (AngII) activation of the angiotensin II type 1 receptor (AT1R). AngII/AT1R activation has also been linked to inflammation-mediated cardiovascular pathologies, where most notably, excess AngII has been implicated as the causative agent leading to increased oxidative stress, vascular fibrosis, and atherosclerosis [14] (Figure 1). In contrast, activation of the “non-classical” or protective arm of RAS through the increased biosynthesis of angiotensin peptides such as Ang(1-9), Ang(1-7), Ang(1-5) and alamandine (AAng(1-7)) may be able to counterbalance the inflammatory response through activation of angiotensin II type 2 receptor (AT2R), Mas receptor (MasR) and Mas-related G-protein coupled receptor (MrgD), respectively. The activation of these receptors has been associated with anti-inflammatory and vasodilatory responses systemically [15].

The molecular mechanism by which AngII/AT1R-promote inflammation includes increased NADPH oxidase expression to produce reactive oxygen species (ROS), which in turn can activate nuclear factor kappa B (NF-kB) [16]. This signaling cascade elicits a transcriptional response in which pro-inflammatory genes such as IL-6, IL-8 and TNF-alpha are upregulated. AngII/AT1R induced oxidative stress can also mediate endothelial dysfunction and apoptosis. Moreover, ROS generation is a key component in initiating the atherosclerotic disease process via oxidative modification of lipoproteins in the intimal layer and initiation of inflammation within vessel walls. Low-level chronic inflammation in turn may contribute to atherosclerotic complications such as ischemia, acute coronary syndromes, and stroke [17].

Although AngII is a key element in promoting atherosclerotic mechanisms, there is little evidence to date as to its role in HIV-related CVD. Less studied still are the effects of angiotensin III (AngIII) and angiotensin IV (AngIV) in relation to HIV-related CVD in humans (Figure 1). AngIII activation of the angiotensin II type 2 receptor (AT2R) has been observed in cardiovascular pathology and mediates vasodilation while preventing vascular remodeling [18]. In contrast, AngIV exerts both pro-atherogenic and anti-atherogenic responses dependent upon where AngIV localizes [19,20]. However, these observations were all made in the absence of chronic viral infections such as HIV. Here, we utilize a metabolomics approach to quantify the levels of known RAS peptides, comparing them in HIV-positive and HIV-negative individuals to determine the difference in virally suppressed individuals. In addition, we explore the association between angiotensin peptides, inflammatory cytokines, and carotid intimal thickness (CIMT), a marker of arterial injury without regard to HIV status.

## 2. Methods

### 2.1. Study Participants

A cross-sectional analysis of HIV-positive and HIV-negative adult participants enrolled in a study evaluating the role of monocyte/macrophage in HIV-related CVD risk was conducted. This study was approved by the University of Hawaii Committee on Human Subjects and conducted according to the criteria set by the declaration of Helsinki. Informed consent was obtained from all participants in the study. The study enrolled adults, age > 40 years old with documented HIV infection who were virally suppressed (HIV RNA PCR < 50 copies/mL) and controlled on suppressive ART for at least 3 months. This group of individuals was compared to the HIV-negative group.

### 2.2. Clinical Assessment

General medical history with special emphasis on CVD and HIV infection was obtained. Clinical parameters including blood pressure (BP), height, weight, body mass index (BMI), and waist-to-hip ratio were measured. Smoking was defined as a dichotomous variable of ever smoked versus never smoked. Blood tests, including CD4 + T-cell count, HIV RNA PCR, fasting (nothing by mouth for 12 h) total, high-density lipoprotein (HDL), directly measured low-density lipoprotein (LDL) cholesterol, triglycerides, and glucose were performed within 3 weeks of CIMT procedure. Hypertension was defined as systolic BP >140 mm Hg, or diastolic BP >90 mm Hg on entry visit, self-reported history of hypertension, or the use of antihypertensive medications. Diabetes mellitus was defined by fasting blood sugar (FBS) of 126 mg/dL or self-reported history of diabetes mellitus. Ten-year coronary heart disease (CHD) risk was calculated by Framingham risk score (FRS) based on a model comprised of gender, age, cholesterol and systolic pressure [21]. Undetectable plasma HIV RNA was defined as HIV RNA 50 copies/mL or less.

### 2.3. RAS Peptide Metabolites

Plasma specimens were collected in a 500X Protease inhibitor cocktail and stored at −80 °C until analysis. Duplicate plasma samples were collected and analyzed within 3 months following collection. The level of angiotensin peptides such as Angiotensin(1-12) [Ang(1-12)], Angiotensin(1-10) [Ang I)], Angiotensin(1-9) [Ang(1-9)], Angiotensin (1-8) [Ang II], Angiotensin(1-7) [Ang(1-7)], Angiotensin(2-8) [AngIII], Angiotensin(3-7) [Ang(3-7)], Angiotensin(3-8) [AngIV], and Angiotensin(1-5) [Ang(1-5)] were quantified using a validated liquid chromatography–mass spectrometry assay performed in the University of Southern California School of Pharmacy. This assay has a lower level of quantification (LLQ) of 10 pg/mL.

### 2.4. Plasma Biomarker Assessment

Stored plasma aliquots were tested for the following analytes: Plasma aliquots were thawed and prepared following kit manufacturer guidelines. Briefly, custom Luminex panels (R&D systems, MN, USA) were run according to the following: CCL2, Galectin-9, soluble (s)CD163, interleukin-6 (IL-6), D-dimer, and tumor necrosis factor alpha (TNFα) were run as a 6-plex with undiluted plasma; C-reactive protein (CRP) and soluble (s)CD14 were run at a 1:200 plasma dilution. Data were acquired on a Luminex 200^TM^ analyzer (Luminex) and analyzed using MILLIPLEX^®^ Analyst software (Millipore). ELISAs for Oxidized low-density lipoprotein (LDL; Mercodia, NC, USA) and Neopterin (Neopterin competitive enzyme immunoassay, ALPCO, NH, USA) were run according to the manufacturer’s instructions. Optical density was read with a microplate spectrophotometer (Bio-Rad). ELISA data were interpolated using a four-parameter logistic curve carried out using the online MyAssays Ltd (Brighton, Cornwall, UK). Data analysis tool. Average intra-assay coefficient of variation (CV) and inter-assay CV for each analyte ranged from 3.94 to 8.32%. All samples were analyzed in duplicate.

### 2.5. Carotid and Bifurcation Carotid Artery Intima-Media Thickness

Ultrasound images of the right carotid artery using a high-resolution, single, B-mode technique were acquired at the Queen’s Medical Center in Honolulu. The analysis of the common carotid artery intima-media thickness (CIMT) was performed at the University of Southern California Atherosclerosis Research Unit Core Imaging and Reading Center using automated edge detection to measure the CIMT of the far wall of the distal common carotid artery (CCA) and right carotid bifurcation (BIF). Right-sided ultrasound images were taken as set forth by USC Atherosclerosis Research Unit’s protocol. To determine whether CIMT is associated with these RAS peptide biomarkers independent of HIV status, analyses using linear regression and stepwise variable selection-based predictive models were developed between independent variables and RCCA or RBIF as the dependent outcome variable.

### 2.6. Statistical Analyses

Demographic data were reported using means and standard deviations for continuous variables and frequencies/percentages for categorical variables. Subject characteristics were compared between HIV-positive and HIV-negative groups using independent t-tests for continuous variables and Chi-Square tests for categorical variables, respectively. Angiotensin peptide levels were collected as continuous variables and summarized using medians and interquartile range (IQR) due to the skewness in variable distributions. The angiotensin peptide ratios were calculated using the product-to-substrate ratio as a surrogate marker of the corresponding aggregate enzyme activity. Wilcoxon rank sum test was performed to compare angiotensin peptide levels between HIV-positive/negative groups. Using the linear regression and stepwise variable selection process, predictive models were developed for right common carotid artery (RCCA) and right bifurcation (RBIF) outcome variables, by taking angiotensin variables and subject characteristics as predictors. A two-sided P-value of less than 0.05 was considered statistically significant. Data analysis was conducted using SPSS software (IBM, Version 24, Armonk, NY, USA) and Prism (GraphPad, Version 8.0, San Diego, CA, USA).

## 3. Results

### 3.1. Clinical Demographics

Patient demographics are summarized in Table 1, where the mean age and sex were compared between the HIV-positive and HIV-negative groups. In this study, hypertension was defined as systolic BP >140 mm Hg, or diastolic BP >90 mm Hg on entry visit or self-reported history of hypertension. Usage of antihypertensive medications irrespective of indication is listed separately. There was a higher number of hypertensive individuals in the HIV-positive group, however, this difference was not statistically significant. The two groups were well balanced with regard to demographics and physiological parameters except for BMI in the male participants, which was significantly higher in the HIV negative group (*p* < 0.01). Although not statistically significant, the individuals in the HIV-positive group had higher cholesterol as compared to the HIV-negative group with *p*-value of 0.07.

### 3.2. Systemic RAS Metabolites

Table 2 summarizes the plasma angiotensin peptide levels presented as median with IQR. In this analysis, HIV-positive individuals had higher levels of all angiotensin peptides quantified except for Ang(1-12), Ang(1-5) and AA(1-7). To determine the overall metabolic formation rate of the angiotensin peptides, a product/reactant ratio was calculated for the various metabolites (Table 3). HIV-negative individuals had a higher product/reactant ratio for AngII/AngI (*p* = 0.03) and Ang(1-5)/Ang(1-7) (*p* = 0.001) when compared to HIV-positive individuals. In contrast, HIV-positive individuals had a higher Ang(1-7)/AngII as compared to their HIV-negative counterpart (*p* = 0.03). Within the HIV-positive participant subset, eleven out of seventy-one individuals had taken a protease inhibitor as a part of their ART regimen. Additional analyses revealed the administration of protease inhibitors did not significantly alter RAS peptide levels in PLWH.

Since antihypertensive agents such as angiotensin-converting enzyme inhibitors (ACEi) and angiotensin receptor blockers (ARB) exert their pharmacological activities by altering RAS peptide binding onto receptors and/or metabolism, a sub-analysis excluded all individuals in both groups receiving antihypertensive medication(s) (Table 4). This reduced the number of individuals to 58 and 40 for the HIV-negative and HIV-positive cohorts, respectively. There were significantly more HIV-positive individuals receiving antihypertensive medication than HIV-negative (*p* < 0.001). A summary of this comparison is highlighted in Table 4. This showed that HIV-positive individuals continued to have statistically higher levels of most angiotensin peptides as compared to the HIV-negative group. When individuals on anti-hypertensive medications were excluded from the analysis, AngII levels were significantly higher in the HIV-positive group by four-fold (*p* = 0.04). Similar to the entire group analyses, precursors (Ang(1-12), AngI, and Ang(1-9)) for AngII were all significantly higher (*p* < 0.03) in the HIV-positive group.

### 3.3. Intimal Media Thickness Association

Predictive association models were developed with either RCCA or RBIF as the dependent outcome variable. Clinical variables, plasma cytokine levels (Table 5), and RAS peptide levels were added to the model through stepwise regression. These analyses showed HIV status alone has no association with RCCA or RBIF. Regression analyses revealed that age, tumor necrosis factor alpha (TNF-alpha), AngII, and AngIII were associated with RCCA (*p* < 0.001) (Table 6). It also showed that AngI/Ang(1-12) ratio (*p* = 0.005) and TNF-alpha (*p* < 0.002) were associated with RBIF. In both RCCA and RBIF models, a statistically significant negative association was observed with AngIII (*p* < 0.03).

## 4. Discussion

This study used a comprehensive RAS metabolomics approach to identify potential mechanisms that may increase cardiovascular risk in HIV-positive individuals. Overall, HIV-positive individuals had statistically higher plasma angiotensin peptide levels as compared to HIV-negative, with the exception of Ang (1-12), AA(1-7) and Ang(1-5). In this study, HIV-positive individuals had 13-fold higher AngI levels as compared to HIV-negative. This change may be able to alter the formation of downstream bioactive RAS peptides. Levels of AngII were 3.5-fold higher in the HIV-positive group, which was accompanied by 1.8-fold increases for both Ang(1-9) and Ang(1-7) as well. In contrast, HIV-negative levels of Ang(1-5) were 3-fold higher as compared to their HIV-positive counterpart. Since antihypertensive agents such as ACEIs and ARBs exert their pharmacologic effect by altering RAS receptor interaction and/or metabolism, an analysis excluding all individuals on antihypertensive medication was undertaken. In these analyses, the HIV-positive patients had a 4.3-fold higher AngII (*p* = 0.04), which was now statistically significant when compared to HIV-negative individuals. More importantly, the exclusion of individuals on antihypertensive medications did not alter the findings from the entire cohort where the HIV-positive group had higher median levels of angiotensin peptides except for Ang(1-5) as compared to HIV negative group.

A number of angiotensin peptides are AT1R agonists, including Ang(1-12), AngII, and AngIII (Figure 1). AngII/AT1R binding is well established and can activate profibrotic wound healing, vasoconstriction, and the induction of inflammation, where its elevation has been associated with increased cardiovascular risk in both HIV-negative and HIV-positive individuals [22,23]. As a weaker agonist than AngII, AngIII has less vasopressor activity (≈30–40% of AngII) but is equipotent to AngII in stimulating the biosynthesis and release of aldosterone from adrenal glands or vasopressin from the brain. Ang(1-12) exerts its effect on the AT1R to enhance vasoconstriction and cell growth in the cardiovascular system [24,25]. Similar to the entire cohort analysis, this sub-analysis showed that fold changes of AT1R agonists such as Ang(1-12) and AngII, were balanced by fold increases in relation to Ang(1-9) and Ang(1-7). All of these changes may be due to increased expression of AngI levels found in the HIV-positive group. While there is a balance between AngII and Ang(1-12) with Ang(1-9) and Ang(1-7), the HIV-positive group had a deficit in terms of Ang(1-5), a MasR agonist, where the HIV-negative group had 3-fold higher levels. Ang(1-9), Ang(1-7), and now Ang(1-5) have been shown to promote atrial natriuretic peptide (ANP) secretion via the MasR [26]. HIV-negative individuals also had a higher Ang(1-5)/Ang(1-7) ratio which was 1.8-fold higher than the HIV-positive group. It is still unclear whether lower levels of Ang(1-5) play a vital role in exacerbating cardiovascular diseases among the HIV-positive population. In animal models, Ang(1-5) was shown to augment atrial natriuretic peptide (ANP) in a dose-dependent manner [24]. ANP has various effects on blood pressure regulations and fluid-electrolyte balance. It is possible that chronic deficiency in ANP may lead to hypertensive disease and other sodium-retaining disorders such as congestive heart failure.

Using a metabolomics approach, we have identified elevated Ang(1-12) levels based on the distribution in HIV-positive patients for both analyses including and excluding individuals on antihypertensive medication. Ang(1-12) is an AT1R agonist whose activity can be antagonized by either ARB or ACEi. [27]. Moreover, Ang(1-12) has vasoconstrictor activity similar to AngII as shown in rat arteries extending from the core to the periphery [28]. To further affirm the role of Ang(1-12), antibodies directed against the C-terminal of Ang(1-12) were able to induce immediate blood pressure reduction [29]. Chan et al. showed that Ang(1-12) was able to increase blood pressure but required a significantly higher concentration as compared to AngII. In vitro studies confirmed that Ang(1-12), whose EC_50_ is 0.19 to 28.7 nM (290 pg/mL to 43.3 ng/mL) to activate AT1R, which was 300-fold less potent as compared to AngII. Unlike AngII which can also activate angiotensin II receptor type 2 (AT2R), Ang(1-12) is unable to activate AT2R [25].

In this analysis, HIV status had no significant association with RCCA or RBIF. Association analyses between RCCA or RBIF with age, RAS peptides, and cytokine parameters revealed that increased RCCA thickness was associated with TNF-alpha, AngII, and AngIII. Similar to other reports, this study supports the role of TNF-alpha as a pro-atherogenic cytokine [21,22]. AngII/AT1R is linked to NF-kB activation which regulates the expression of TNF-alpha and other inflammatory cytokines, a concept that is also supported by the current study. Association between AngIII, a less potent AngII agonist of AT1R, revealed a negative association with CIMT for both RCCA and RBIF [30]. This finding is consistent with ELISA measurements of AngIII showing lower levels associated with coronary atherosclerosis [19,20,24]. Although AngIII is an AT1R agonist, its inverse relationship with CIMT suggests potential competition between AngII or Ang(1-12) with AngIII to activate AT1R, a concept that needs to be experimentally confirmed. Moreover, AngIII is also an AT2R agonist promoting vasodilation while preventing vascular remodeling which may partially explain the negative correlation [18]. RAS peptide ratio analyses showed that AngI/Ang(1-12) is associated with RBIF. Higher circulating levels of Ang(1-12) in the HIV-positive suggest a non-RAS enzyme may mediate its biosynthesis. Chymase is produced by cardiomyocytes which are associated with tissue remodeling in atherosclerosis [31]. Ang(1-12) is expressed in the left ventricle of the heart in hypertensive and normotensive rats [32]. Elevated levels of Ang(1-12) may also serve as a source to form additional AngI and AngII through chymase or angiotensin-converting enzyme 1 (ACE1)-mediated metabolism [32,33]. This set of data taken together shows that Ang(1-12) is a bioactive angiotensin peptide that can promote AT1R-mediated activities which may contribute to vasoconstriction and increase RBIF.

Higher Ang(1-12) and AngII levels found in HIV-positive individuals may contribute to the pathogenic arm of RAS. However, HIV-positive individuals also had elevated Ang(1-9) and Ang(1-7), members of the protective arm of RAS that were significantly higher in the HIV-positive group by 1.8-fold. Both peptides are byproducts of angiotensin-converting enzyme 2 (ACE2)-mediated metabolism. This may suggest a counterbalance of high levels of AngII and Ang(1-12) through the upregulation of components of the protective arm of RAS in HIV-positive individuals. This study suggests that virally suppressed HIV-positive individuals have higher levels of RAS peptides in the protective arm of RAS, which may counterbalance higher levels of AT1R agonists such as Ang(1-12) and AngII.

The findings of this study identify changes in RAS metabolism in HIV-positive individuals. These metabolites serve as potential biomarkers for the physiological assessment of carotid thickness irrespective of HIV status. Limitations of this study include a lack of specificity regarding the enzyme kinetics implicated in peptide metabolism. Whether these conversions are driven by ACE-mediated enzymes or alternative enzyme pathways such as neprilysin (NEP), prolyl endopeptidase (PEP), or chymase was not determined. However, the activity of each of these enzyme preparations has been determined by Rice et al. via active-site titrations using competitive tight-binding inhibitors [34].

## 5. Conclusions

Using a metabolomic approach that is capable of quantifying the levels of known RAS peptides, this study affirmed the importance of AngII and TNF-alpha as proatherogenic factors associated with RCCA and RBIF that was independent of HIV status. The lack of association with HIV positivity may be due to prolonged period of viral suppression. The relationship between AngII and TNF-alpha has been established through the activation of NF-kB. Our comprehensive approach also showed that HIV-positive individuals had compensatory increases in Ang(1-7) and Ang(1-9), both of which are members of the protective arm of RAS in response to increased levels of AT1R agonists such as Ang(1-12) and AngII. Two unexpected findings include the role of Ang(1-12) and AngI/Ang(1-12) ratio revealing these parameters as potential pro-atherogenic factors, and reduction in Ang(1-5) in the HIV-positive group. Although the exact mechanism of how these peptides relate to the overall increase in CIMT of the RCCA and RBIF is unknown, this study shows the utility of quantitative RAS metabolomics in dissecting molecular pathways associated with atherosclerosis. Additional studies will be necessary to confirm whether these changes are associated with HIV infection or ART, which has also been linked to atherosclerosis.

## Figures and Tables

**Table 1:** Patient demographic data.

Variable	HIV Negative (*n* = 72)	HIV Positive (*n* = 71)	*p* Value
Age	58.96 (9.29)	59.36 (7.59)	0.931
Sex (M), *n* (%)	61 (84.7)	61 (84.7)	1.000
Ethnicity, White, *n* (%)	37 (51.4)	44 (61)	0.278
Height, in	67.78 (3.62)	68.45 (3.58)	0.265
Weight, lbs.	193.10 (54.47)	181.30 (36.94)	0.130
BMI (M), kg/m^2^	29.83 (7.52)	26.94 (4.05)	0.009
BMI (F), kg/m^2^	26.89 (6.53)	27.55 (5.28)	0.804
Systolic blood pressure, mmHg	131.9 (17.47)	125.2 (13.42)	0.011
Diastolic blood pressure, mmHg	78.14 (10.88)	77.18 (10.19)	0.589
Mean arterial pressure, mmHg	96.05 (11.87)	93.17 (10.02)	0.121
Hypertension, *n* (%)	24 (33.3)	31 (43.1)	0.255
Antihypertensive medication use, *n* (%)	13 (44%)	32 (18%)	<0.001
High Cholesterol, *n* (%)	25 (34.7)	36 (50)	0.074
Diabetes, *n* (%)	6 (8.3)	9 (12.5)	0.429
Framingham risk score (%)	13.5 (13.1)	12.1 (9)	0.460
CD4 count cells/mm^3^	-	673 (311)	-
Presence of carotid plaque, *n* (%)	25 (34.7)	32 (45.1)	0.260
Current tobacco smoking, *n* (%)	8 (11%)	8 (11%)	0.976
Substance use, including alcohol, *n* (%)	52 (72%)	67 (93%)	0.002
Beta blocker use, *n* (%)	7 (9.7)	7 (8.9)	0.978

Values reported as mean with standard deviation or counts with percentages. *p*-values were determined using independent two sample t-tests for continuous variables and Chi-square for categorical variables.

**Table 2:** Circulating plasma RAS peptide levels of all patients at study entry.

RAS Peptide	HIV Negative N = 72	HIV Positive N = 71	*p*-Value	RAS Peptide Median Ratios (HIV+/HIV−)
	Median [IQR] [ng/mL]	Median [IQR] [ng/mL]		
Ang(1-12)	0.05 [0.01–0.05]	0.05 [0.03–0.50]	<0.001	1.00
AngI (Ang(1-10))	0.05 [0.01–0.40]	0.67 [0.05–2.14]	<0.001	13.40
Ang(1-9)	0.34 [0.06–0.63]	0.60 [0.19–1.04]	0.002	1.76
AngII (Ang(1-8))	0.11 [0.05–0.48]	0.38 [0.05–0.74]	0.223	3.45
Ang(1-7)	0.05 [0.02–0.26]	0.09 [0.05–0.42]	0.016	1.80
Ang(1-5)	0.33 [0.05–0.60]	0.09 [0.05–0.37]	0.003	0.27
AA(1-7)	0.05 [0.01–0.53]	0.05 [0.01–0.33]	0.755	1.00
AngIII (Ang(2-8))	0.53 [0.29–0.64]	0.61 [0.11–1.53]	0.092	1.15
AngIV (Ang(3-8))	0.10 [0.05–0.44]	0.28 [0.05–0.67]	0.019	2.80
Ang(3-7)	0.05 [0.02–0.10]	0.10 [0.05–0.19]	0.005	2.00

Variables were summarized using median with interquartile range (IQR). *p*-values were determined using the Wilcoxon rank-sum tests. RAS peptide ratio is reported as the ratio of medians (HIV+/HIV−).

**Table 3:** Plasma RAS peptide ratios (product/reactant) at study entry.

RAS Peptide Ratio (Product/Reactant)	HIV Negative N = 72	HIV Positive N = 71	*p*-Value	Median Ratios (HIV+/HIV−)
	Median [IQR]	Median [IQR]		
AngI/Ang(1-12)	2.0 [1.0–9.83]	1.0 [0.70–7.69]	0.102	2.00
Ang(1-9)/AngI	1.0 [0.49–19.61]	1.30 [0.48–13.0]	0.495	1.30
AngII/AngI	1.0 [0.15–11.96]	0.56 [0.24–1.0]	0.028	0.56
Ang(1-7)/Ang(1-9)	0.42 [0.05–0.89]	0.22 [0.06–0.57]	0.624	0.52
Ang(1-7)/AngII	0.76 [0.14–1.0]	0.86 [0.56–1.0]	0.034	1.13
Ang(1-5)/Ang(1-7)	1.87 [1.0–11.15]	1.0 [0.28–5.67]	0.001	0.54
AA(1-7)/Ang(1-7)	1.0 [0.26–7.99]	1.0 [0.11–2.31]	0.248	1.00
Ang(3-7)/Ang(1-7)	0.89 [0.20–1.0]	0.66 [0.30–1.0]	0.761	0.74
AngIII/AngII	1.89 [1.28–4.45]	2.11 [1.23–8.72]	0.368	1.12
AngIV/AngIII	0.40 [0.11–1.0]	0.38 [0.10–1.0]	0.905	0.95
Ang(3-7)/AngIV	0.84 [0.14–1.27]	0.45 [0.29–1.07]	0.679	0.54

Variables were summarized using median with interquartile range (IQR). *p*-values were determined using the Wilcoxon rank-sum tests. Ratios calculated as product peptide concentration/precursor peptide concentration. Fold change in RAS peptide level reported as HIV-positive/HIV-negative ratio.

**Table 4:** Circulating RAS peptides in HIV-negative versus HIV-positive individuals not on antihypertensive medications.

RAS Peptide	HIV Negative N = 58	HIV Positive N = 40	*p*-Value	Median Ratios (HIV+/HIV−)
	Median [IQR]	Median [IQR]		
Ang(1-12)	0.05 [0.01–0.05]	0.05 [0.01–0.88]	0.013	1.00
AngI (Ang(1-10))	0.05 [0.05–0.36]	0.47 [0.05–1.89]	0.004	9.40
Ang(1-9)	0.30 [0.05–0.63]	0.59 [0.19–1.02]	0.024	1.97
AngII (Ang(1-8))	0.10 [0.05–0.49]	0.43 [0.05–0.77]	0.039	4.30
Ang(1-7)	0.05 [0.05–0.24]	0.18 [0.05–0.47]	0.013	3.60
Ang(1-5)	0.34 [0.05–0.64]	0.11 [0.01–0.53]	0.048	0.32
AA1-7	0.05 [0.01–0.52]	0.05 [0.01–0.50]	0.451	1.00
AngIV(Ang(3-8))	0.12 [0.05–0.46]	0.30 [0.06–0.69]	0.017	2.50
Ang(3-7)	0.05 [0.01–0.10]	0.1 [0.05–0.22]	0.003	2.00
AngIII (Ang(2-8))	0.45 [0.07–0.63]	0.62 [0.32–3.67]	0.015	1.38

Values reported as median with interquartile range. *p*-values were determined using Wilcoxon rank-sum tests.

**Table 5:** Patient plasma cytokine levels.

Cytokine	HIV Negative N = 72	HIV Positive N = 71	*p*-Value
	Median [IQR]	Median [IQR]	
Neopterin	7.18 [4.23–8.82]	9.45 [7.49–11.68]	<0.001
CD14	1.64 × 10^6^ [1.26 × 10^6^–2.26 × 10^6^]	1.94 × 10^6^ [1.56 × 10^6^–2.47 × 10^6^]	0.012
CRP	1.319 × 10^6^ [4.73 × 10^5^–3.18 × 10^6^]	8.75 × 10^5^ [4.25 × 10^5^–2.35 × 10^6^]	0.130
TNF alpha	1.27 [0.10–2.41]	2.39 [1.57–3.29]	<0.001
IL-6	0.75 [0.46–1.10]	1.11 [0.82–1.66]	<0.001
CCL2	85.13 [71.07–99.56]	94.00 [78.04–114.70]	0.022
CD163	1.21 × 10^5^ [7.52 × 10^4^–1.61 × 10^5^]	1.38 × 10^5^ [9.39 × 10^4^–2.21 × 10^5^]	0.034
D-dimer	796.6 [597.6–628.2]	967.9 [628.2–1650]	0.204

Values reported as median (pg/mL) with interquartile range. D-dimer concentrations reported in ng/mL. *p*-values were determined using Wilcoxon rank-sum tests.

**Table 6:** Multivariate linear regression model for biomarkers associated with carotid thickness.

Dependent Variable	Model Variables	Beta Coefficient	Sig. (*p*-Value)
Right Common Carotid Artery Thickness (RCCA)	(Constant)	0.432	0.005
	Age (years)	0.003	0.030
	HIV positive	−0.019	0.511
	Gender (Female)	−0.004	0.906
	MAP (mmHg)	0.001	0.430
	TNF alpha (pg/mL)	0.022	<0.001
	AngII (ng/mL)	0.208	<0.001
	AngIII (ng/mL)	−0.026	<0.001
Right Carotid Bifurcation Thickness (RBIF)	(Constant)	0.499	0.004
	Age (years)	0.003	0.071
	MAP (mmHg)	0.002	0.171
	Gender (Female)	−0.027	0.488
	HIV positive	−0.009	0.789
	AngI/Ang(1-12)	0.001	0.005
	TNF alpha (pg/mL)	0.016	0.002
	AngIII (ng/mL)	−0.014	0.029

Each model was adjusted for age, HIV status, gender, and mean arterial pressure (MAP).

## Data Availability

The data presented in this study are available on request from the corresponding author due to protect patient privacy.

## Nonstandard Abbreviations and Acronyms

RAS	renin–angiotensin system
RCCA	right common carotid artery
RBIF	right carotid bifurcation
CIMT	carotid intima-media thickness
AT1R	angiotensin II type 1 receptor
AT2R	angiotensin II type 2 receptor
ACE1	angiotensin-converting enzyme 1
ACE2	angiotensin-converting enzyme 2
NEP	neprilysin
PEP	prolyl endopeptidase
ANP	atrial natriuretic peptide
